# Delivering on the promise of better health for women, children, and adolescents

**DOI:** 10.1080/16549716.2025.2542609

**Published:** 2025-09-19

**Authors:** Austin Demby, Luc Laviolette

**Affiliations:** a Minister of Health, Government of Sierra Leone, Freetown, Sierra Leone; b Global Financing Facility, World Bank, Washington, USA

**Keywords:** Global Financing Facility for Women, Children and Adolescents: Examining National Priorities, Processes and Investments, Global financing facility, health systems, women, children, adolescents

## Abstract

This commentary is included in the Special Series: *Global Financing Facility for Women, Children and Adolescents: Examining National Priorities, Processes and Investments*. Studies in the Series provide timely, grounded insights into how the GFF model delivers impact through country ownership, flexible funding, and integrated planning. The Series’ findings align with the recent independent evaluation of the GFF, emphasizing the importance of political leadership, implementation support, learning systems, and civil society engagement. The commentary reflects on the lessons emerging from the papers in the Special Series and examines what they mean for strengthening accountability, systems, and equity in the GFF’s next strategy.

## Paper context

- **Added knowledge**: this commentary adds perspective on the Global Financing Facility policy series, rather than new knowledge per se
- **Global health impact for policy and action**: this commentary adds a perspective on impactful global health issues addressed in the Global Financing Facility policy series

## Background

The Global Financing Facility (GFF) emerged from a critical recognition during the transition from the Millennium Development Goals to the Sustainable Development Goals: advancing the health and rights of women, children, and adolescents required not just increased funding, but smarter, better-coordinated financing mechanisms led by the countries they aimed to support.

Established as part of the Every Woman Every Child architecture and championed by the UN Secretary-General, the GFF was designed to align with this new vision. By empowering countries to take charge of their health plans and by coordinating both domestic and international resources in support of those plans, the GFF can prevent the deaths of women and children, reduce stillbirths, and significantly improve population health.

Through Investment Cases (ICs) that align external and domestic resources around priority interventions, countries set their own health agendas. By integrating in-country technical support and small volumes of catalytic trust fund grants with larger financing streams such as the World Bank’s International Development Association (IDA) grants and low-interest loans, the GFF delivers resources needed to address reproductive, maternal, newborn, child, adolescent health and nutrition (RMNCAH-N) challenges.

As a recent independent evaluation [1] of the GFF affirmed, ‘The unique value of the GFF lies in its ability to bring together different approaches essential to delivering sustainable RMNCAH-N results with a focus on strengthening country leadership through government-led country platforms and Investment Cases (ICs) that prioritize RMNCAH-N investments and strengthen health systems.’ [2]

The findings from the independent evaluation are backed up by the perspectives of Ministers of Health in GFF partner countries, who place great value in GFF’s flexible, integrated systems strengthening approach and catalytic, on-budget investment model, which is anchored in a strong commitment to country leadership and alignment.

At a time when global health financing faces unprecedented challenges – from donor retrenchment and domestic economic constraints to shifting priorities post-COVID19 pandemic – the need for the GFF’s flexible, country-driven platform has never been greater. Initiatives, such as the Lusaka Agenda, that aim to sharpen the focus on sustainable health financing, country leadership, and systems resilience, align with all areas where the GFF plays a vital role.

## Why the global health action collection matters

We welcome the Global Health Action Special Series, published from June 2024, as another timely, independent contribution to understanding how the GFF model delivers impact in practice – particularly as the GFF develops its strategy beyond 2025. In-depth external analyses, particularly when rooted in on-the-ground realities, are essential to the continuous evolution of any complex development initiative. Global Health Action’s Special Series provides both granular country-specific narratives and broader thematic analyses of how GFF-supported programs have been implemented and experienced.

Many of the Special Series’ conclusions from last year align with the recent independent evaluation, underscoring the importance of this kind of evidence in shaping the GFF’s future strategy. Rather than viewing these analyses in isolation, we see them as complementary to the GFF learning agenda and integral to informing the GFF’s next five-year strategy, currently under development.

## Country-centered learning

The GFF’s core value lies in its country-led approach. ICs are developed through national platforms that bring together ministries of health and finance, donors, civil society, and technical partners. The process is iterative by design and results in a shared set of priorities that guide the alignment of financing and implementation efforts.

The Special Series’ country-specific analyses illustrate both the promise and the complexity of this approach. They also align, in many areas, with the 10 country case studies conducted by the independent evaluation of the GFF, which highlighted, among other topics, the value of flexible implementation approaches and strong political leadership, the need for robust systems to capture and share lessons learned across countries, and a call for more sustained engagement, including with civil society. The GFF is committed to implementing enhanced learning systems that capture insights as they emerge, enabling more timely course corrections and better knowledge sharing across the portfolio. This will strengthen the GFF’s ability to support countries while continuously improving the model.

## Cross-cutting themes and what comes next

The Special Series’ thematic papers on adolescent sexual and reproductive health, maternal and newborn health, community health, and policy analysis offer valuable perspectives on cross-cutting implementation challenges. Their findings complement the independent evaluation’s [1] documentation of results across thematic areas.

Both analyses, the GFF independent evaluation and the Global Health Action Special Series, point to the GFF’s growing contribution to health system strengthening, improved service delivery, and enhanced financial management, while acknowledging areas where the GFF must continue to strengthen its approach and demonstrate impact. As the GFF develops its next five-year strategy, these thematic insights can help inform how the GFF enhances its support for countries’ highest-priority interventions. The GFF is particularly focused on strengthening the articulation of its contribution to improved health outcomes, while ensuring the GFF approach remains responsive to evolving country needs and global health priorities.

## Conclusion: from evidence to action: refining the GFF model for greater impact and ambition

The GFF is not static. Since its inception, it has evolved in response to country experiences, evidence, and shifts in the global health and development landscape. This evolution will continue, and accelerate, as the GFF finalizes the next five-year strategy.

Independent research, such as the GHA’s Collection, plays an essential role in supporting GFF’s process of continual refinement in response to country realities. The GFF is committed to translating these insights into action, deepening support for countries, improving accountability for results, and remaining relentlessly focused on the mission.

In today’s constrained funding environment, with growing needs and heightened urgency, the value of the GFF model is more evident than ever. Country-driven. System-focused. Equity-oriented. The GFF remains committed to advancing health and rights for women, children, and adolescents – because their future is our shared future – and health is the foundation for wider progress.
